# Medical Inspection of Schools

**Published:** 1900-07-14

**Authors:** 


### MEDICAL INSPECTION OF SCHOOLS.

The London School Board has spent the last two Thursday afternoons in debating whether it will try, as an experiment in two districts, the preventive visitation of schools, and has finally shelved the matter by "referring back." Now there can be no doubt that schools do spread infection.

Dr. Shirley Murphy, medical officer of health to the London County Council, says in his report of 1898 on diphtheria and elementary schools: "The influence of schools in disseminating infectious disease has been long recognised, and in respect of diphtheria was shown, as early as 1876, by Mr. W. H. Power. There is, therefore, no improbability in the suggestion that, with increasing school attendance and corresponding increase of opportunity of infection at school, the result should be manifested in increased incidence of diphtheria mortality upon children at the school age. The figures contained in this report show that this increased incidence has actually occurred, and, although some part of it is probably attributable to change in nomenclature, there is, I submit, a substantial balance due to this greater opportunity of infection at school."

Dr. R. J. Neece, in his report to the Local Government Board of 1895 says: "School attendance has been the means of spreading diphtheria in Flint."

Dr. S. W. Wheaton, in his report of 1897, says: "With regard to the spread of diphtheria after the disease had once become established in Wrexham, there is abundant evidence to show that this occurred chiefly by personal infection, and mainly by personal infection received while attending school."

Dr. Theodore Thomson, in his report on the spread of measles at Burton-on-Trent in 1898, says: "As regards the manner of propagation of this epidemic, investigation led to the conclusion that schools had materially contributed thereto."

Indeed, all the reports on this subject furnished by the Local Government Board come to the same conclusion. Take also the following figures for London as to the mean annual rate of mortality per 1,000 (England and Wales being taken as 100) in three infectious diseases, remembering that Forster's Education Act was passed in 1871:—

| | 1871-80. | 1881-90. | 1891-98. |
|---|---|---|---|
| Diphtheria | 101 | 160 | 207 |
| Measles | 134 | 143 | 143 |
| Scarlet fever | 83 | 100 | 115 |

Then as regards the lesser but not less evil contagions, we have the evidence of some of our London Board School teachers and school nurses that 90 per cent. of children in poorer schools have been found to be suffering from parasitic diseases or contagious eye disease; and Dr. Arnold Lea, in his paper before the Blackpool Health Congress last September, said: "Pediculi and parasitic diseases are so common that in some schools over 70 per cent. of the children have been found to be infected. There is also a large amount of distress caused by children with early phthisis, chorea, epilepsy, &c., being forced to attend school." That is the case as it stands; it practically means that in poor districts compulsory education means compulsory contagion of the most uncleanly and disgusting kind, and compulsory disease.

What is the remedy? Last year's Medical Congress passed a resolution requesting the British Medical Association to take steps towards obtaining power for medical officers of health to inspect elementary schools.

Dr. Shirley Murphy says: "The circumstance that schools contribute largely to the prevalence of this disease not only justifies but necessitates the medical officer of health having free access to the schools, where school children can be most readily examined, for the prosecution of his inquiries."

Dr. Arnold Lea says: "I would suggest that some such scheme of daily medical inspection of our Board schools is urgently required in this country. I see among my out-patients at a large children's hospital a number of children each week who continue to attend school whilst suffering from infectious and parasitic disease, from refractive errors, and many other affections. I am convinced that school infection plays a considerable part in spreading contagious diseases, and it is our duty to protect the children whilst attending school to the fullest extent possible. The rich and well-to-do have solved the question years ago. At all our great public schools a doctor is in daily attendance, and suspicious cases are at once isolated and investigated."

Dr. Windle, in his report of 1898 on diphtheria in Southall, says: "It is impossible to speak too highly of the benefits which have accrued from this systematic examination of the school children in stamping out the disease. But to be a preventative measure in the true sense of the word, it is necessary that such an examination should be carried out in non-epidemic times, and especially is it necessary on the re-assembling of scholars after holidays, for it was then that we found most cases to occur. Even if such a limited systematic examination were instituted throughout the country, I feel convinced that not only would there be a remarkable diminution of diphtheria, but other infectious diseases, as measles, scarlet fever, whooping cough, &c., spreading by personal contact, would decline in like proportion."

And if we look to other countries, we find in New York, Boston, Berlin, Liepsic, Brussels, Chicago, and many other towns that the Educational Authorities have already started preventive visitation of schools. Obviously the sanitary authorities of London must take up the work, and place it in the hands of medical officers appointed for the purpose. London cannot afford to have infection spread, because the London School Board employs one medical officer to run round and close schools after an epidemic is well established.

The following is the suggestion that has been shelved: "That the Board hereby sanctions in principle, as an experiment for two years, in certain schools in the Tower Hamlets and Southwark Divisions, a system of preventive visitation; and that for this purpose the Board authorise the temporary engagement of two medical men, at a salary of £250 per annum each, and six nurses, at a salary of £80 per annum each—one medical man and three nurses being allotted to each of the sets of selected schools; and that it be referred to the General Purposes Committee to make the necessary arrangements."

The employment of nurses as well as medical men was urged, because of the good work done already by the school nurses, because it would be a pity to waste a medical man's time on dirty heads, and because a New York inspector of schools says his work is practically useless for aught save exclusion, and is actively effective so far as a voluntary nurse assists him.

We have quoted from medical men, let us now quote what Government officials have to say. Her Majesty's inspector in his report of April 19th, 1900, on Laxton Street School, writes: "The visits of a nurse to this very large infants' school have proved most beneficial to the health of the children, so much so that it could be wished that the School Board might make such visits universal in their schools in poor localities." And A. Eichholtz, M.B. and H.M.I., when giving evidence before the School Board's sub-committee on infectious diseases, spoke strongly of the good work done by school nurses in his district, but also advocated the employment of local medical officers to direct the nurses and undertake the more serious examinations.

Obviously the Board of Education is far ahead of the School Board on this subject. But the community cannot be left to suffer because of the opinions of the School Board in this matter. "The health of the children is the capital of the nation" the Bishop of London said at the annual meeting of the School Nurses last week; and however grateful we are for that voluntary society which is showing the nation the way, our "capital" cannot be left in the hands of a small irresponsible body who are hampered for funds. The School Board and the sanitary euthorities should between them arrange for the preventive visitation of elementary schools in London.

